# Deep learning synthesis of cone-beam computed tomography from zero echo time magnetic resonance imaging

**DOI:** 10.1038/s41598-023-33288-8

**Published:** 2023-04-13

**Authors:** Hyeyeon Choi, Jong Pil Yun, Ari Lee, Sang-Sun Han, Sang Woo Kim, Chena Lee

**Affiliations:** 1 Department of Electrical Engineering, Pohang University of Science and Technology, 77 Cheongam-ro Nam-gu, Pohang, 37673 Republic of Korea; 2 Daegyeong Division, Korea Institute of Industrial Technology, Daegu, Republic of Korea; 3 Department of Oral and Maxillofacial Radiology, Yonsei University College of Dentistry, 50-1 Yonsei-ro Seodaemun-gu, Seoul, 03722 Republic of Korea; 4 Institute for Innovative in Digital Healthcare, Yonsei University, Seoul, Republic of Korea

**Keywords:** Oral anatomy, Dental radiology

## Abstract

Cone-beam computed tomography (CBCT) produces high-resolution of hard tissue even in small voxel size, but the process is associated with radiation exposure and poor soft tissue imaging. Thus, we synthesized a CBCT image from the magnetic resonance imaging (MRI), using deep learning and to assess its clinical accuracy. We collected patients who underwent both CBCT and MRI simultaneously in our institution (Seoul). MRI data were registered with CBCT data, and both data were prepared into 512 slices of axial, sagittal, and coronal sections. A deep learning-based synthesis model was trained and the output data were evaluated by comparing the original and synthetic CBCT (syCBCT). According to expert evaluation, syCBCT images showed better performance in terms of artifacts and noise criteria but had poor resolution compared to the original CBCT images. In syCBCT, hard tissue showed better clarity with significantly different MAE and SSIM. This study result would be a basis for replacing CBCT with non-radiation imaging that would be helpful for patients planning to undergo both MRI and CBCT.

## Introduction

The innovation of cone-beam computed tomography (CBCT) has revolutionized the aspect of treatment in dentistry. CBCT is preferred over multi-slice CT (MSCT) in the dental field because it uses micro-unit-sized isotropic voxels, minimizing image distortion when reconstructed in non-orthogonal directions, such as the tooth axis or cross-section of the dental arch^1^. The high-resolution aspect of CBCT, compared to MSCT, also facilitates the assessment of minute structures in the desired direction^2,3^. However, CBCT images cannot analyze soft tissue density due to poor contrast with artifacts and noise. In cases when it is necessary to diagnose both soft and hard tissue pathology, such as in temporomandibular disease, magnetic resonance imaging (MRI) should be acquired for soft tissue diagnosis and CBCT.

Radiation-free imaging, especially magnetic resonance imaging (MRI), is advancing rapidly with the recent introduction of bone MRI sequences. Hilgenfield et al.^4,5^ reported that MRI-based implant planning was reliable and sufficiently accurate. Although the diagnostic accuracy and reliability of bone MRI has been shown to be equivalent to those of CT, the unfamiliar contrast and a lot of imaging noise are the limitations for the immediate application of this technique for clinical conditions^6^. Due to such limitations, there have been several studies to convert head and neck MRI into CT images using deep learning^7–11^. Yet, the current literature suggests MRI transformation models based on MSCT images and there is no study based on CBCT as an imaging source.

In previous studies for the MRI to MSCT conversion, the U-Net was widely used as a baseline for synthesis networks; Wang et al.^11^ and Bahrami et al.^12^ showed the capability of U-Net on MSCT synthesis. Han et al.^8^ and Massa et al.^9^ used modified U-Net architecture inspired by the VGG network and Inception module, respectively, which have shown excellent feature extraction capabilities in computer vision tasks. Generative model-based methods were also utilized in previous studies^13^. Gholamiankhah et al.^7^ compared the quality of samples generated from ResNet and generative adversarial networks. Qi et al.^10^ utilized conditional GAN with multi-channel inputs from head and neck MRI. Regarding CBCT images, none attempted to use MRI as a source image, but a few studies used MSCT images as a source. Yuan et al.^14^ utilized MSCT images as a source to synthesize CBCT using U-Net to reduce the artifact distortion of CBCT. Conversely, Chen et al.^15^ utilized CBCT as an input of cycleGAN to generate MSCT images with enhanced scattering artifacts. Unlike existing studies, the synthesis of CBCT from MRI requires additional consideration of registration errors and different patterns of each modality. In this article, we proposed a novel method for CBCT synthesis based on previous studies.

Therefore, we hypothesized that a deep learning model would synthesize CBCT images accurately from MRI images, specifically adjusted to describe hard tissue, including tooth and alveolar and maxillofacial bones. We attempted to synthesize accurate CBCT images using various pre-processing MRI methods. In addition, it was assumed that the synthesized images could be utilized for 3D modeling, similar to the conventional CBCT images used in clinics.

## Materials and methods

### Ethics

This study was approved by the institutional review board (IRB No. 2-2021-0027) of Yonsei University Dental Hospital and was conducted and completed in accordance with the ethical regulations. Due to the retrospective nature of the study, the requirement for informed consent was waived and this was approved by Yonsei University Dental Hospital, IRB. All imaging data were anonymized before export.

### Data collection

For this study, 21 patients who underwent both CBCT and MRI for temporomandibular joint (TMJ) disease in our institution were randomly selected and examined. The CBCT-MRI paired data set was randomly divided into training (n = 16) and test (n = 5) sets.

All CBCT images were obtained with an Alphard 3030 unit (Asahi Roentgen, Kyoto, Japan) using the following parameters: tube voltage, 90 kVp; tube current, 8 mA; exposure time, 17 s; field of view (FOV), 150 × 150 mm; and voxel size, 0.3 mm. There was no modification on the reconstruction filter from the projection into the axial image data and the default parameters provided by the manufacturer were used in this study.

MRI was performed using the 3.0 T scanner (Pioneer; GE Healthcare, Waukesha, WI, USA) with a 21-channel head coil. Isotropic three-dimensional zero echo time (ZTE) sequences were acquired with the following parameters: TE/TR, 0/785 ms; flip angle, 4°; receiver bandwidth, 31.25 kHz; number of excitations (NEX), 2; FOV, 180 × 180 mm (supra-orbital rim to upper neck region); acquisition matrix, 260 × 260; voxel size, 0.35 mm; slice thickness, 1.0 mm; and scan time, ~ 5 min.

### Data preparation

Paired image data were registered due to differences in patient orientation during image acquisition. The entire registration process was conducted via ITK-snap (ver. 3.0, www.itksnap.org). The gross orientation of MRI (anterior–posterior position) was matched with CBCT orientation (superior-inferior position) manually. Then, based on the mutual information, geometrical rigid registration was conducted until the mutual information between the two images reached its maximum^16^.

Then, the MRI image was resliced into the same thickness, 300 $\upmu$m, as the CBCT image. Five hundred and twelve CBCT and MRI axial slides were prepared. For data augmentation, axial image data were reconstructed into 512 coronal and sagittal slides each, and all images were prepared in the BMP format. In total, 64,512 images (3,072 images per data pair) of CBCT and MRI data were prepared.

### Deep learning network training

**Figure 1:**
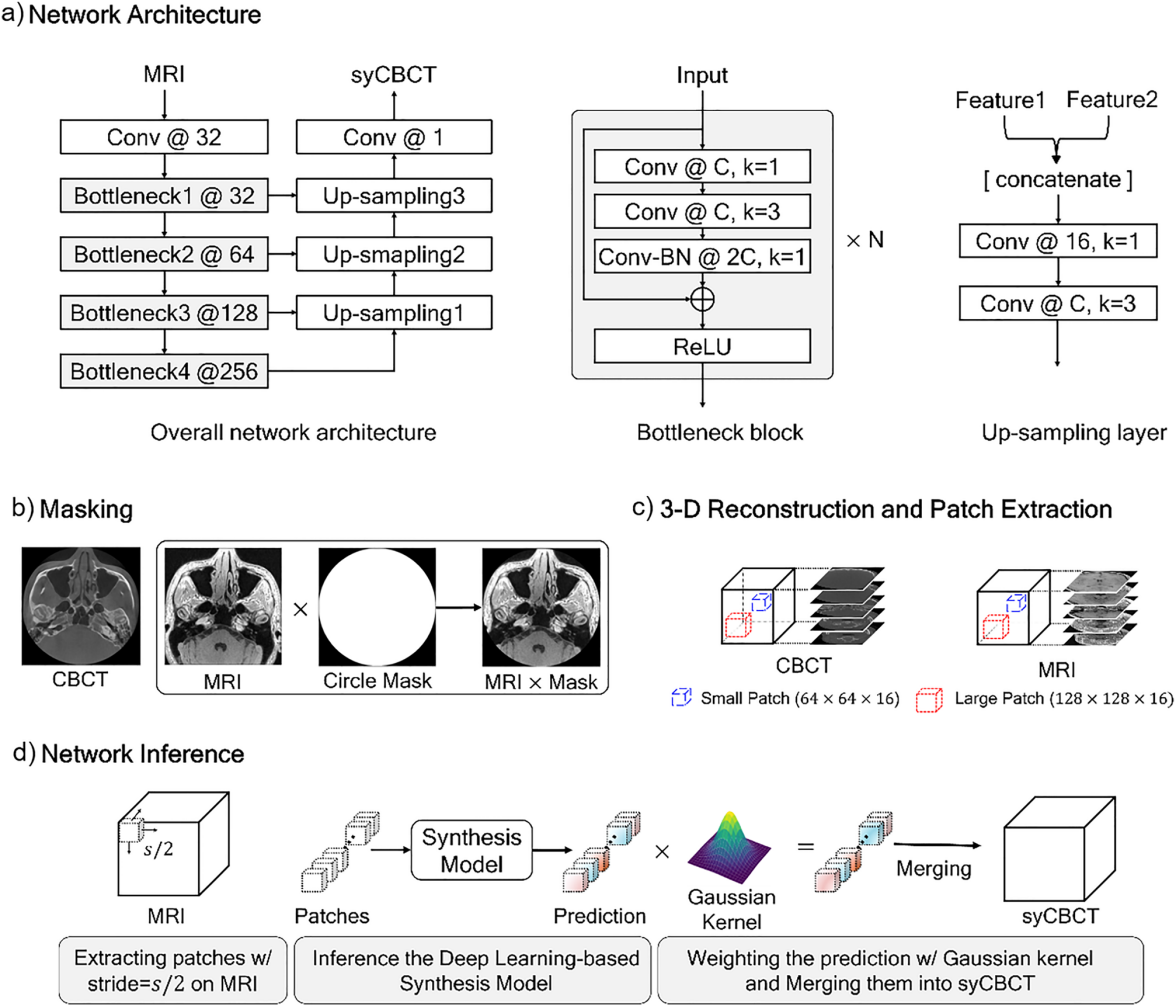
The overall network training and inference process. (**a**) Our network architecture. Conv denotes the kernel series of [Convolution-Batch Normalization (BN) -Rectified Liner Unit (ReLU)], N indicates the number of repeated bottleneck blocks, the number after the symbol @ represents the number of kernels, and k denotes the kernel size. (**b**) The MRI and CBCT are multiplied by a circle mask at the pixel level. (**c**) Large or small patches are extracted from the 3-dimensional reconstructed image. (**d**) An input MRI is partitioned into sub-patches, and then predictions of the trained network are weighted by the Gaussian kernel.

A modified U-Net structure was used for our synthesis model. U-Net is commonly applied to biomedical imaging tasks, as it shows relatively higher accuracy than existing networks with a small number of source images^17^. To enhance the result performance by extracting more hierarchical features than those of the original U-Net, we modified several parts of the network structure as illustrated in Fig. 1. First, the encoder structure was substituted with the Bottleneck blocks of ResNet-50^18^, and all 2-dimensional convolution layers were changed into 3-dimensional convolution layers. Second, the last skip connection of the U-Net was removed because the minute registration error between MRI and CBCT makes the morphology of synthesized prediction confusing, and the different patterns of the input MRI can affect the results. Lastly, to prevent the model capacity from exceeding our hardware memory size, the number of convolution kernels was changed, as described in Fig. 1a. The ablation studies for each proposed component were performed.

Sixteen sets of MRI-CBCT pairs were used for training the synthesis model, and five sets were evaluated as test sets. For pre-processing, we multiplied the MRI-CBCT pair by a circle binary mask with a radius of 256 pixels to remove the background noise (Fig. 1b). Then, the masked images were stacked in the vertical direction to reconstruct a 3-D image of size 512 × 512 × 512. Due to different field of view size, peripheral area loss occurs in specific images of MRI and CBCT sequences. The noisy sequences were excluded in the training step to ensure stable network training. We used only 21–490, 1–360, and 41–380 sequences for the x, y, and z axes of the entire image, respectively. To overcome the limitation of the hardware (memory size) and execute the data augmentation, we randomly extracted patches from the whole image. The experiments were conducted with two different sizes of patches; a large patch of size 128 × 128 × 16 and a small patch of size 64 × 64 × 16, as illustrated in Fig. 1c.

The network was trained by Adam optimizer with an initial learning rate of 2.5 × ${10}^{-4}$, that was exponentially decayed by 0.8 every 200 iterations, and the weight decay was 10^–5^. The smooth L1 loss and the early stopping method were used with a stopping factor of 5. The mini-batch sizes were 32 and 8 for the small and large patches, respectively. The input patches were normalized to [− 1, 1].

In the inference phase, an input MRI image was partitioned into the patches using a sliding window method, with the step size being half the patch size (Fig. 1d). The trained synthesis model predicted the CBCT patches. Then, each patch was weighted by the Gaussian filter to generate a smooth cross-section of 3-D synthetic CBCT (syCBCT). Finally, the syCBCT was merged by overlaying weighted patches with the same stride of the sliding window.

### Accuracy assessment and clinical validation

#### Three-dimensional model surface deviation

**Figure 2:**
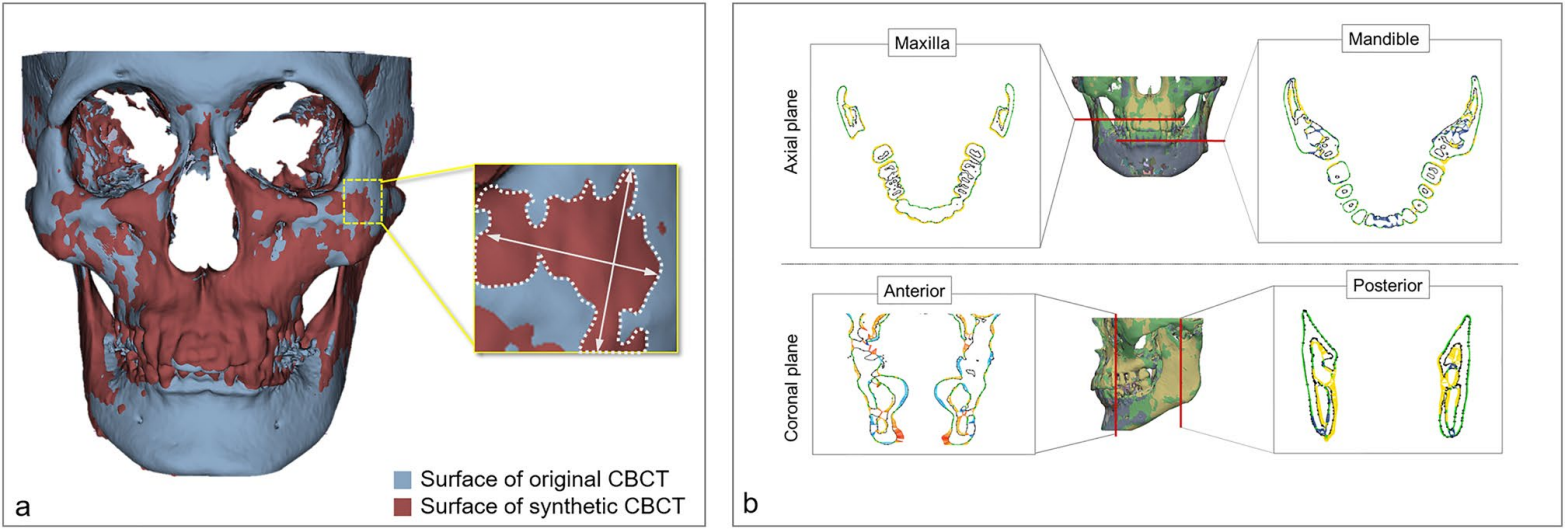
Surface deviation of synthetic CBCT. (**a**) The deviation of the overall three-dimensional model. (**b**) The deviation measurement in axial (maxilla and mandible) and coronal (anterior and posterior) planes. The black line indicates the surface of the original CBCT, and the colored line indicates deviated area. CBCT, cone-beam computed tomography; syCBCT, synthetic CBCT.

A three-dimensional maxillofacial model was generated in the STL format based on both original CBCT and syCBCT. The two models were superimposed for measurement using Geomagic Control X (3D Systems, Cary, NC, USA). Then, the overall surface deviation was acquired (Fig. 2a) for both large and small patches based on syCBCT. The surface deviation of syCBCT was also obtained for anatomical regions, maxilla, and mandible in the axial and anterior–posterior coronal planes (Fig. 2b). The reference planes were determined by following a previous study^19^. The axial plane was determined by the cement o-enamel junction of the upper and lower teeth. Anatomical landmarks, including mental foramen (anterior) and mandibular foramen (posterior), were used to determine the coronal plane. All measured deviation values were obtained in root mean square (RMS, mm).

#### Expert image quality evaluation

Two radiologists with more than 10 years of experience conducted a subjective evaluation using the modified version of the clinical image evaluation chart of CBCT provided by the Korean Academy of Oral and Maxillofacial Radiology (Table 1). The clinical image evaluation chart comprises 4 sections: artifact, noise, resolution, and overall image. In the artifact, noise, and resolution sections, the evaluator graded image series as poor, moderate, or good. For overall grade, the possible outcomes were: no diagnostic value, poor, moderate, or good.

**Table 1:** Clinical image evaluation chart of CBCT.

	Grade			
	Poor	Moderate	Good	
[Artifact] 1. Streak artifact 2. Ring artifact 3. Blurring edge and shading				
[Noise] 4. Anatomic structure region 5. Background				
[Resolution] 6. Enamel, dentin, pulp 7. PDL space and lamina dura 8. Sinus floor 9. Bone pattern 10. TMJ complex				
	Grade			
[Overall image]	No diagnostic value	Poor	Moderate	Good

CBCT, cone-beam computed tomography; PDL, periodontal ligament; TMJ, temporomandibular joint.

#### Image quality evaluation metrics

For five sets of test data, the image quality of the syCBCT in axial series was compared to that of the original CBCT image using three indices, mean absolute error (MAE), peak signal-to-noise ratio (PSNR), and structural similarity indexing method (SSIM), that are frequently used to evaluate synthetic images^20^. MAE suggests a correlation with the image noise level, PSNR is closely related to the clarity and resolution of the image, and SSIM is comprehensively correlated with the structural similarity of the synthetic image. The definition and ideal reference value^18^ of each index were as follows:

1 $$MAE = \frac{1}{N}\mathop \sum \limits_{i = 1}^{N} \left| {syCT_{i} - CT_{i} } \right|,\;{\text{reference}}\;{\text{value}} = 0\;{\text{HU}}$$

2 $$PSNR = 10 \times {\text{log}}\left( {\frac{{f_{max}^{2} }}{{rmse^{2} }}} \right),\;{\text{reference}}\;{\text{value}} > {25}\;{\text{dB}}$$

3 $$SSIM = \frac{{\left( {2\mu_{{\hat{y}_{i} }} \mu_{{y_{i,k} }} + c_{1} } \right)\left( {2\sigma_{{\hat{y}_{i} y_{i,k} }} + c_{2} } \right)}}{{\left( {\mu_{{\hat{y}_{i} }}^{2} + \mu_{{y_{i,k} }}^{2} + c_{1} } \right)\left( {\sigma_{{\hat{y}_{i} }}^{2} + \sigma_{{y_{i,k} }}^{2} + c_{2} } \right)}},\;{\text{reference}}\;{\text{value}} = {1}$$

All metrics were obtained according to the ability to present hard tissue, soft tissue, and air in syCBCT compared to the original CBCT^20^.

### Statistical analysis and comparisons

To measure the surface deviation of large and small patch-based 3-D models, RMS values were compared using the Mann–Whitney test. The deviation at the anatomical regions (maxilla, mandible, posterior, and anterior) was compared using the Kruskal–Wallis test and Dunn’s multiple comparison post-hoc test. The number of grades from the clinical CBCT image evaluation chart according to each criterion (artifact, noise, resolution, and overall) was also assessed for original CBCT and syCBCT images. Inter-observer agreement was obtained by interclass correlation coefficient (ICC). The image quality metrics, MAE, PSNR, and SSIM, were compared for hard and soft tissue as well as air in individual syCBCT using one-way ANOVA. Statistical analysis was conducted with GraphPad Prism version 9.4.1 (GraphPad Software, La Jolla, CA, USA, www.graphpad.com) and a confidential interval of 95%.

## Results

**Figure 3:**
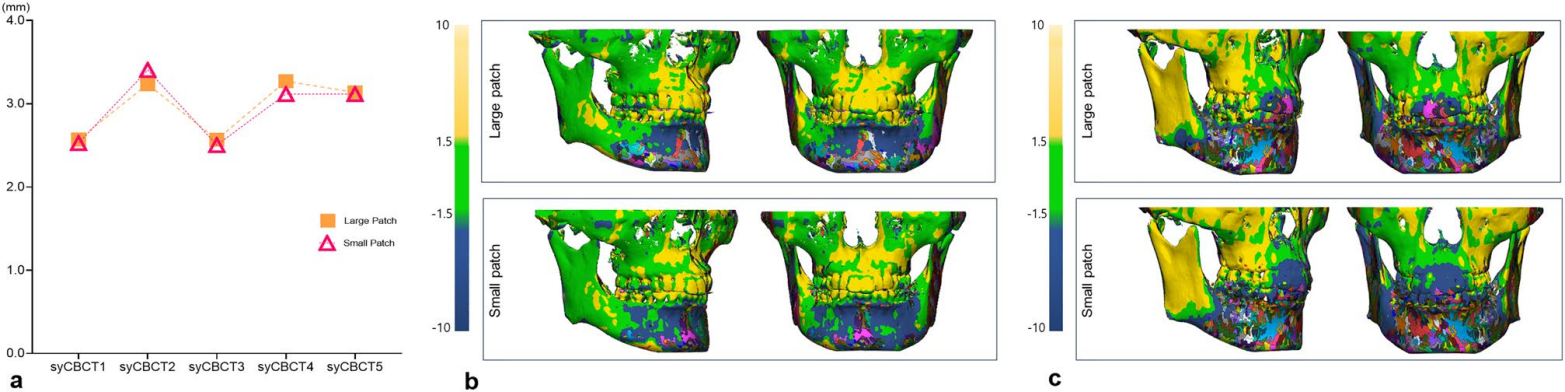
(**a**) The overall surface deviation of three-dimensional models ranged from 2.5 to 3.4 mm. (**b**) Surface deviation color maps in 3D models showing the smallest deviation (syCBCT 3). (**c**) Surface deviation color maps in 3D models show the largest deviation (syCBCT 2). CBCT, cone-beam computed tomography; syCBCT, synthetic CBCT.

The mean surface deviation was 2.95 $\pm \hspace{0.17em}$0.35 and 2.93 $\pm \hspace{0.17em}$0.39 mm for large and small patch-based syCBCT, respectively, and there was no statistical difference. Four small patch-based 3D models showed less surface deviation than large patch-based models, while one small patch-based 3D model (syCBCT2) showed more surface deviation than large patch-based models (Table 2, Fig. 3). In deviation measured at different anatomical regions, the anterior region showed larger deviation (large patch, 3.76 mm; small patch, 4.01 mm), and the maxilla showed smaller deviation (large patch, 3.09 mm; small patch, 2.81 mm) (Table 2). The mean surface deviation between the maxilla and anterior region in small patch-based models was significantly different.

**Table 2:** Mean value of surface deviation in overall three-dimensional models and the respective anatomical region.

(Unit: mm)							
	Overall	*p* value*	Maxilla	Mandible	Anterior	Posterior	*p *value**
Large patch	2.95 ± 0.36	0.52	3.09 ± 0.72	3.43 ± 0.29	3.76 ± 0.41	3.19 ± 0.70	0.23
Small patch	2.93 ± 0.39		2.82 ± 0.67^a^	3.36 ± 0.58^a^	4.01 ± 0.27^b^	3.16 ± 0.80^a^	0.05**

*Mann–Whitney test, 95% confidential interval.

**Kruskal–Wallis test and Dunn’s multiple comparisons post-hoc test, 95% confidential interval.

**Figure 4:**
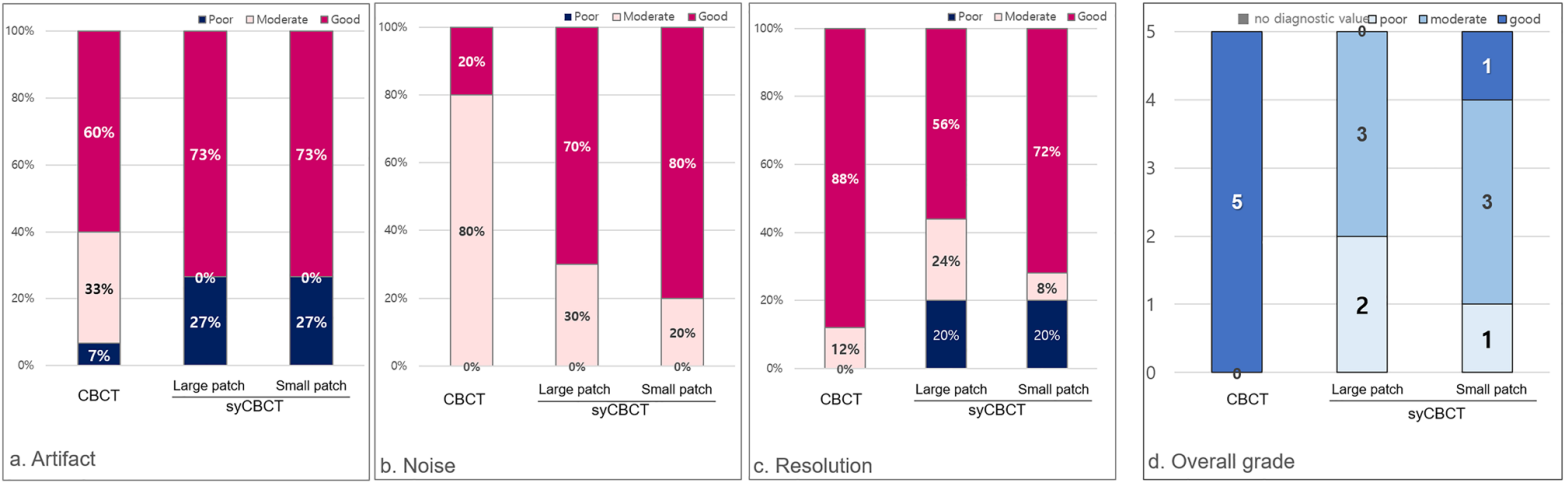
The expert image quality evaluation according to (**a**) artifact, (**b**) noise, (**c**) resolution, and (**d**) overall image grade.

**Figure 5:**
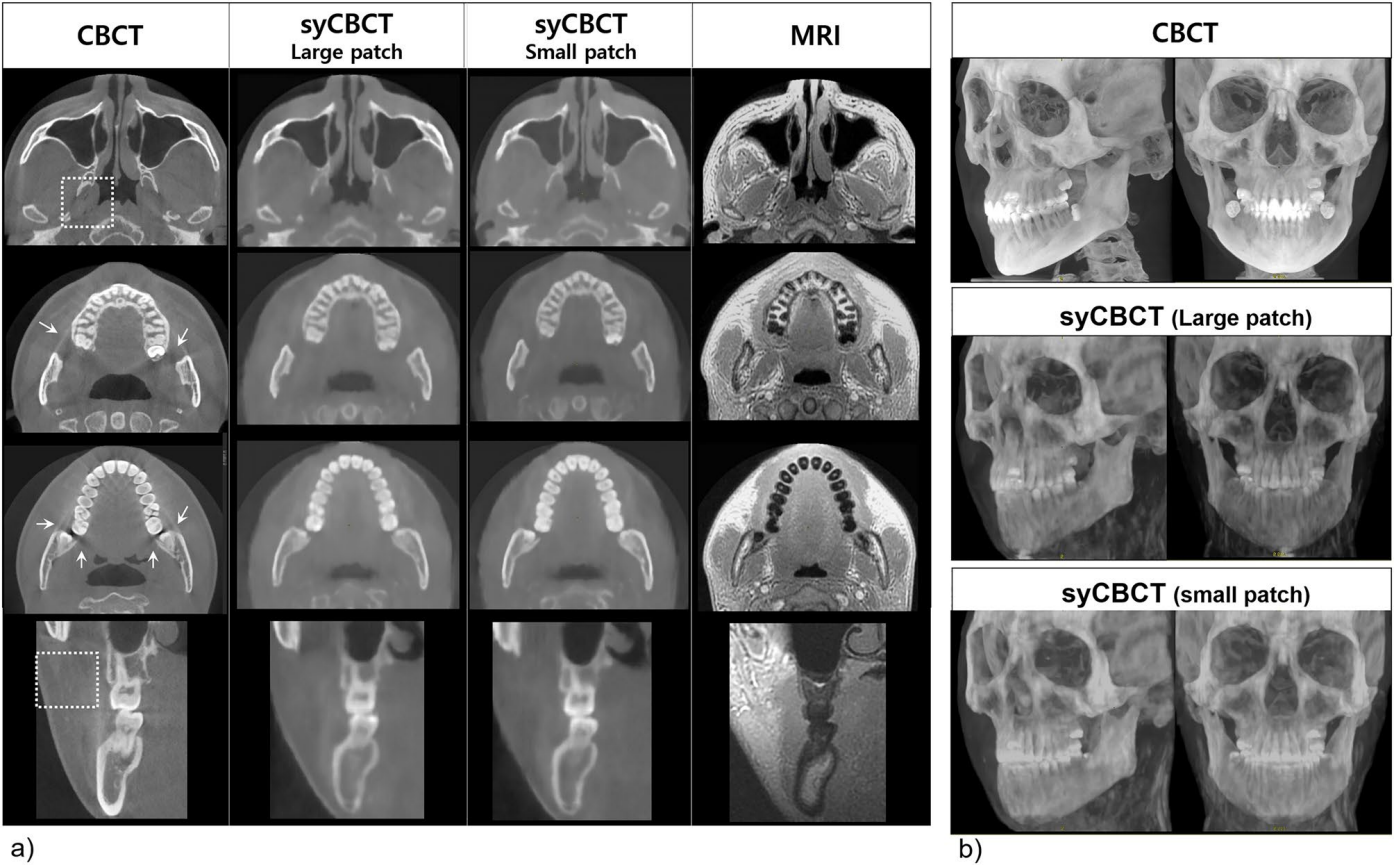
(**a**) The axial and cross-sectional images show the soft tissue of syCBCT with less noise, whereas more heterogeneity is observed in CBCT (dotted box). The scattering artifact (arrow) in the CBCT image is not evident in the syCBCT image. The margin of tooth structure is blurred in syCBCT. (**b**) Three-dimensional reconstruction of CBCT and syCBCT using the maximum intensity projection method. CBCT, cone-beam computed tomography; syCBCT, synthetic CBCT.

Expert image quality evaluation showed that syCBCT provided better performance in terms of artifact and noise criteria than the original CBCT. On the contrary, the original CBCT obtained a ‘good’ grade for the resolution criterion (Figs. 4, 5). All original CBCTs showed a ‘good’ grade for the overall image, while only one syCBCT based on small patch models showed a ‘good’ grade (Fig. 4d). The ICC between the evaluators was 0.85.

The proposed network introduced structural changes based on U-Net and applied a Gaussian filter at post-processing. The ablation studies for each proposed component were performed for the small patch, and the corresponding results are listed in Supplementary Table S1. Among the image quality metrics, MAE and SSIM showed significantly better performance in evaluating hard tissue structures (Table 3). However, PSNR showed the best performance in describing air. All three types of tissues showed significantly different level of image quality according to all indices. Additionally, all indices (except SSIM) showed better performance in small patch-based-syCBCT than in the large patch-based image for hard tissue.

**Table 3:** Image quality evaluation metrics based on hard tissue, soft tissue, and air evaluation of the image.

	Hard tissue	Soft tissue	Air	*p*-value
Mean absolute error				
Large patch	3.41 ± 1.43	23.47 ± 1.94	13.97 ± 4.18	< 0.0001*
Small patch	3.29 ± 1.47	21.06 ± 1.55	12.92 ± 3.77	< 0.0001*
Peak signal-to-noise ratio				
Large patch	19.61 ± 1.60	16.63 ± 0.70	20.46 ± 1.40	< 0.0001*
Small patch	19.73 ± 1.71	17.24 ± 0.78	20.96 ± 1.36	< 0.0001*
Structural similarity index map				
Large patch	0.8944 ± 0.03	0.6847 ± 0.04	0.6183 ± 0.07	< 0.0001*
Small patch	0.8934 ± 0.03	0.6923 ± 0.04	0.6220 ± 0.07	< 0.0001*

*One-way ANOVA with 95% confidential interval.

## Discussion

This study was the first approach to synthesizing dental CBCT images based on ZTEMRI images using deep learning. It is considered an important attempt at this point intime when the need for radiation-free and low-dose dental imaging is increasing. As a result of this study, syCBCT images comparable to CBCT images used at present were achieved. The image quality indices, MAE, PSNR, and SSIM, showed acceptable values in the current study compared to the previous medical image synthetic studies. It was significant that the syCBCT image was superior to the original CBCT image in terms of artifacts and noise, though the resolution was insufficient. In addition, 3D model manipulation, which was challenging based on MRI, showed feasibility through this study.

It was significant that the syCBCT showed improvement in the artifacts and noise of the image compared to the original CBCT. These unexpected results have not been reported in any previous studies on CT image synthesis based on MRI data, probably because all studies focused on multichannel CT rather than CBCT^8,9,11^. Traditionally, compared to multichannel CT, CBCT is known to produce images with extensive noise and artifacts due to a low radiation dose and cone-shaped beam. Many researchers have tried to reduce scattering noise and artifacts in CBCT since its introduction in dentistry^21,22^. Although due to a different phenomenon, MRI also produces highly noisy images with artifacts. Thus, we did not expect to obtain improved syCBCT from MRI in terms of noise and artifacts. This result would have significant potential for research on artifact and noise elimination in CBCT, which has been an unsolved problem up to the present time.

Clinical imaging evaluation depicted that the resolution of syCBCT was unsatisfactory in this study. The original CBCT showed a good to moderate grade of resolution, while syCBCT showed poor to moderate grade resolution. This was consistent with imaging quality metrics. The value of PSNR, which represents the clarity of the image, was less than that in previous similar studies^23^. Among several suspected reasons, relatively low sharpness of the hard tissue structure in MRI could be considered primarily. Although the voxel size and slice thickness of the original MRI data was within the range of clinically used CBCT unit, the relatively low sharpness of the bone margin was considered to be an insurmountable problem of the imaging modality itself. This part needs to be supplemented with the development of additional advanced image post-processing techniques.

Meanwhile, the image noise and artifacts level showed enhanced quality, showing lower MAE values, compared to those in the previous studies^23^. Also, the value of SSIM in our study, which indicates overall image quality, was comparable to that of the previous studies^23^. Although the clarity of the syCBCT image was low in the current study, the overall image quality was comparable to that of previous studies due to reduced noise and artifacts.

The blurred margins and low sharpness of anatomic structures in synthetic CT images have been an issue in deep-learning-based CT image synthesis^7,14,24^, and a similar tendency was shown in our study. Leynes et al.^24^ mentioned that gross bone depiction in syCBCT was comparable to that in the original CT image, whereas it was difficult to depict finer bone structures. Han^7^ also reported that the error in syCBCT mainly occurred at the border of bone tissue. Yuan et al.^14^ studied the production of synthetic CT from fast-scan CBCT based on deep-learning models and stated that small fine details were not preserved in synthetic CT images. The overall resolution of synthetic CT was poorer than that of the original CBCT image. To overcome such a problem, Chen et al.^15^ pre-processed multichannel CT using the up-sampling method. Through this pre-processing, multichannel CT images were turned into images with higher resolution. Accordingly, the synthetic image output was expected to show improved sharpness and clarity. It is mentioned that, despite their efforts, deformation still tends to appear in the output image^15^.

In the training step, we adopted two different-sized patches as input. In the case of small patches, we expected more precise results with less distortion than those in large patches, enabling us to concentrate more on the delicate morphology of the small region. As a result, improved performances were obtained in the image quality metrics, surface deviation, and expert image quality evaluation. However, the statistical differences were not significant. Thus, advanced research about image pre-processing that enhances the sharpness of input images is needed. In addition, we suggest that excluding patches that contain registration errors due to postural differences in the training step will help to improve the quality of syCBCT. Further, one of the issues with comparing surface deviation in the 3D maxillofacial model, was that the model file contains errors due to the conversion of the file type from the original image format. Therefore, the few millimeters deviations should be considered as due to comparing the relative error according to the input data types and different facial regions, and so it is difficult to view as an absolute error.

Chen et al.^15^ mentioned misregistration of the image sets as a possible reason for the synthetic image deformation. The current study included the registration between MRI and CBCT. In particular, the MRI images used in this study could not be completely registered with CBCT images owing to differences in the patient posture during both imaging procedures. Additionally, the MRI used in this study was for TMJ evaluation, and the image signal of the lower submental area, which was relatively far below the TMJ, was not satisfactorily sensitive for accurate model training. Hence, a prospective study design should be established to develop deep-learning models that can synthesize more accurate CBCT images.

Here, a modified U-Net structure with a backbone of ResNet was used. Gholamiankhah et al. and Bahrami et al. compared GAN, eCNN, U-Net, and V-net with ResNet and concluded that ResNet showed the best performance in CT synthesis from MRI^7,12^. We also adapted the ResNet, to take advantage of the feature extraction capability, and removed the last skip connection in U-Net to reduce the disturbance of inevitable registration errors in our dataset. We confirmed that each component of the proposed method improved the quality of syCBCT by conducting the ablation studies (see Supplementary Table S1). Although, all indices did not show best performance, the SSIM, which is known as close to the human visual perception, showed highest values in the proposed model of the current study. MAE and PSNR of hard tissue was degraded quality in the proposed model compared to the previous studies^8,17^, however, the difference was minute that cannot be detected by naked eye of human (Supplementary Figure S1).

Additionally, comparing our proposed method with the existing methods of U-Net^17^ and Han et al.^8^, the proposed method generally showed superior performance in image quality indices (see Supplementary Table S2). Han et al.^8^ avoided using 3-dimensional convolution filters by warring about of GPU memory limit. We changed the number of kernels in each convolution layer to handle this issue. This modification increased the efficiency of the network capacity by reducing the number of model parameters from 31 million (U-Net) to 10 million (ours).

Previous studies utilized the adversarial learning strategy^7,25^, which trains a synthesis model with a discriminator that tries to distinguish target images as either real or synthesized. However, adversarial learning is known to be challenging to optimize due to “mode collapse,” in which a synthesis model keeps generating identical samples^26^. To prevent mode collapse, we used smooth L1 loss instead of adversarial loss. The smooth L1 loss computes pixel-wise differences between original and synthesized images and is relatively robust to recognize outliers rather than mean squared error loss. In our experiment, the artifact of CBCT is considered the outlier, which shows a larger value than other areas. Therefore, it was thought that the utilization of the smooth L1 loss results in reducing syCBCT artifacts would be effective in this study.

There are several limitations to this study. First, although the sample size used in this study was comparable to that of the previous studies, the more enhanced performance of the model can be achieved with more samples due to the nature of deep learning research. Further research with additional MRI and CBCT data sets would help to increase the accuracy of the synthetic image. Additionally, as mentioned above, due to the difference in the patient's position in MRI and CBCT, perfect registration could not be achieved, leading to errors in CBCT image output. In this study, the registration process was conducted using commercial software, while a more sophisticated approach to the registration procedure is required. Lastly, obtaining MRI source data with high image quality, especially in the mandible area, would show a more improved result than that of the current study. Thus, a solid prospective study design would be required to develop more advanced CBCT synthetic models.

## Conclusion

This study provided the first approach to CBCT synthesis from ZTE MRI, a non-ionizing radiation imaging. Compared to the conventional CBCT image, the generated CBCT image showed a clinically applicable level in dentistry with improved image quality in terms of noise and artifact. The study results would be expected to provide a basis for non-ionizing radiation imaging with improved quality for replacing CBCT for patients planning to undergo both MRI and CBCT simultaneously.

## Supplementary Information

Supplementary Information.

## Data Availability

The data generated and analyzed during the current study are not publicly available due to privacy laws and policies in Korea, but are available from the corresponding author on reasonable request.
