# Supplementary material for: Deep learning synthesis of cone-beam computed tomography from zero echo time magnetic resonance imaging
Source: Sci Rep. 2023 Apr 13;13:6031. doi: 10.1038/s41598-023-33288-8 (PMC10102229; doi:10.1038/s41598-023-33288-8)
Supplement: Supplementary file 1 — Supplementary Information. [file 41598_2023_33288_MOESM1_ESM.docx]

**Table S1**. Effect of each proposed element on syCBCT quality. The bold blue font shows the best scores for each tissue type.

| Options of generation method | | | Evaluation metrics | | | |
| --- | --- | --- | --- | --- | --- | --- |
| Bottleneck | Remove the last skip connection | Post-processing (Gaussian kernel) | Tissue type | MAE | PSNR | SSIM |
| U-Net [14] | - | - | Hard tissue | 3.36 | **20.33** | 0.86 |
|  |  |  | Soft tissue | 29.71 | 12.39 | 0.60 |
|  |  |  | Air | 22.48 | 12.51 | 0.56 |
| U-Net [14] | ✔ | - | Hard tissue | 3.22 | 20.04 | 0.56 |
|  |  |  | Soft tissue | 22.01 | 13.16 | 0.62 |
|  |  |  | Air | 16.66 | 13.21 | 0.58 |
| Proposed | ✔ | - | Hard tissue | **2.93** | 20.23 | 0.87 |
|  |  |  | Soft tissue | 22.88 | 13.37 | 0.65 |
|  |  |  | Air | 20.46 | 13.28 | 0.58 |
| Proposed | ✔ | ✔ | Hard tissue | 3.29 | 19.73 | **0.89** |
|  |  |  | Soft tissue | **21.06** | **17.24** | **0.69** |
|  |  |  | Air | **12.92** | **20.96** | **0.62** |

**Table S2**. Results of comparison experiment with previous studies. The bold blue font shows the best scores for each tissue type.

| Method | Number of network parameters | Tissue type | MAE | PSNR | SSIM |
| --- | --- | --- | --- | --- | --- |
| U-Net [14] | 31.0 M | Hard tissue | 3.36 | **20.33** | 0.86 |
|  |  | Soft tissue | 29.71 | 12.39 | 0.60 |
|  |  | Air | 22.48 | 12.51 | 0.56 |
| Han et al. [9] | 34.9 M | Hard tissue | **3.14** | 20.24 | 0.87 |
|  |  | Soft tissue | 22.74 | 13.08 | 0.64 |
|  |  | Air | 18.20 | 13.21 | 0.58 |
| Proposed | 10.0 M | Hard tissue | 3.29 | 19.73 | **0.89** |
|  |  | Soft tissue | **21.06** | **17.24** | **0.69** |
|  |  | Air | **12.92** | **20.96** | **0.62** |


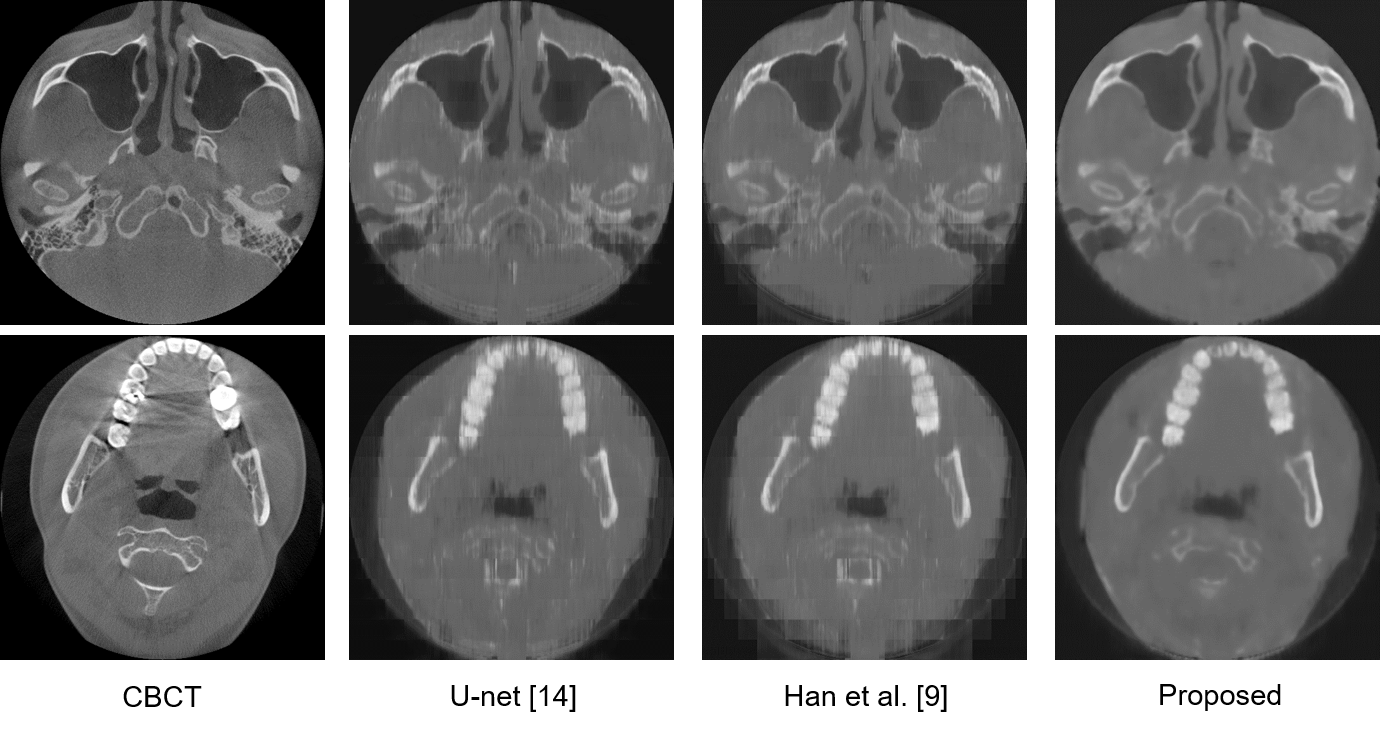


**Figure S1**. Visual comparison of the image quality compared to the previous studies showing superior performance of the proposed model
